# The Blister Treatment of Rheumatic Fever

**Published:** 1866-09

**Authors:** 


*The Blister Treatment of Rheumatic Fever.* Cases under the care of Dr. JEFFERSON, at St. Bartholomew's Hospital.*

The safety of the heart is undoubtedly the main point at which every treatment must be directed; and in this particular more especially does the Blister Treatment exhibit its peculiar value. In a communication read Wednesday, March 22, by Dr. Davis at the Hunterian Society, he stated that of fifty cases which had been admitted under his care at the London Hospital, twenty-seven had hearts already damaged by recent or old inflammatory mischief, and twenty-three were free from cardiac complication. The results of the blister treatment in these fifty cases showed that as many as twenty-five, when discharged from the Hospital, were totally free from any endo- or peri-cardiac disease; or, in other words, that while every heart was saved which came in sound, two recent cases of endo-carditis were apparently cured by the alteration effected, as he believed, in the alkalinity of the blood by the free discharge of serum from the neighborhood of the inflamed joints. Dr. Davis also states that those cases answer best to the treatment in which a great number of joints are simultaneously affected, and when, by setting up, a large amount of discharging surface in the proximity of the inflamed parts, a large

* See last volume of the *Retrospect*, Synopsis, p. 273, for a brief epitome of this plan of treatment.

proportion of the *materias morbi* may be evacuated at one *coup*. Cases where the poison would appear to crop up to the surface by instalments, attacking the various joints at intervals of days, do not afford such striking examples of the efficacy of the treatment. The first case, where an unexampled amount of blister was applied in an extremely acute case, and where the patient was discharged, cured in thirteen days, will well illustrate Dr. Davis's position. That this treatment is not simply local in its action is also shown in the alteration produced in the urine in the majority of the cases cited; for in eleven the urine remained acid, but generally diminished in acidity during the whole period of the case; in twenty-two it became neutral shortly after the serum was discharged; in ten it exhibited alkaline re-action; while in seven no notes were taken.

CASE 1.—William S., aged thirty-two, a working silversmith, and exposed to great variations of temperature, was admitted into the Hospital on December 2nd, the seventh day of his illness, and was discharged cured on December 15th, thirteen days after he came under treatment. Eleven blisters, amounting to 482 square inches, were applied simultaneously, and with almost immediate relief. As the patient said, "the rheumatic pains left me as soon as the blisters drew;" and on the third day from admission all pain had disappeared. The pulse fell from 105 to 95 per minute; the temperature from 101.4 to 99.6 and 98.8; no cardiac mischief was developed. The urine, scanty and acid on admission, was rendered slightly albuminous from the presence in it of a small quantity of blood. The slight stranguary and albumen, however, disappeared in forty-eight hours. He had slept very badly from the commencement of his illness, but as soon as the poultices were applied to the blistered surfaces sleep returned, and was "good" every night during the time he remained in the Hospital. His appetite, bad on admission, was good on the third day; and his thirst, which was slight when he came under treatment, was not increased by the blisters, is reported to be absent on the fourth day. The heart was sound when he came under treatment, and free from disease when he left the Hospital.

CASE 2.—Wm. P., aged twenty-two, bootmaker, was attacked for the first time with rheumatic fever on November 29th. He was admitted into the Hospital on December 6th, the seventh day of his illness, and was discharged on January 2nd, 1865. Seven blisters, equal to 133⅝ square inches, were applied on December 9th, and the pain, which was severe previous to their application, had disappeared as soon as the surfaces had been dressed. The following day the clinical report states: "There is no pain anywhere, and with the exception of a little uneasiness on the right shoulder on December 17, he was quite easy and comfortable until the day of his discharge." The pulse fell from 110 on admission to 90 per minute on the third day. The temperature from 104.4 to 98.6. The heart presented a systolic murmur, distinct at admission over base and apex, and was unchanged at the time of patient's leaving the Hospital. His sleep, which was stated to be bad before the blister application, was improved on the second, good on the third night, and remained satisfactory until he was discharged. The urine presented a slight trace of albumen as the result of the treatment, but was normal and free from that substance forty-eight hours after the blisters had been applied. The appetite, which was indifferent on admission, is reported to have been "good" on the fourth day, while the thirst was absent at first, and continued so, unaffected by the local irritation set up by the blisters.

CASE 3.—John B., aged nineteen, was admitted on December 8, 1866, the seventh day of his disease, and discharged January 30, 1865. He had, however, been up and dressed from January 11, 1865. Eight blisters were applied on December 9, three on the 12th, one on the 13th, one on the 14th, one on the 21st, making in all 603½ square inches of empl. lyttæ. The day succeeding the application of the eight blisters the report states that all pain had disappeared. On the fourth day from that date, the patient has "now a little pain in the right shoulder, and says he wants another blister for it," which is applied and followed by great relief. The clinical account says: "December 14.—The patient has no pain anywhere. December 18.—The same. December 19.—Had pain in the left knee yesterday; I think he caught cold from going to the closet yesterday. And December 20.—The left ankle and knee were swollen." The application of the blister removed all pain and swelling, and never returned during the time he continued under treatment. The pulse fell from 108 to 86, and varied from time to time, with the appearance of the affection in different joints. The temperature fell from 100.8 to 99.4, and also varied with the pulse. The heart presented before the application a systolic murmur at the apex, which remained unaltered at the time of his leaving the ward. His sleep, which had been bad since December 2, was pretty good on the night after the blisters had been dressed; moderate on the fourth; good on the fifth; disturbed by a blister considerably on the 6th; good on the following and every succeeding night. The urine: Its reaction before the blister application was not noted, but the treatment induced some stranguary and traces of albumen, and its condition varied from time to time with the variable condition of the joints. Appetite is stated to have been bad on admission; a little better on the second day from the application of the blisters; improving and gradually becoming good from the fifth day of the treatment. The thirst was slight when the patient entered the Hospital. It appears, however, to have been considerably increased after the application of the blisters, but the symptoms vanished in a few days.

CASE 4.—Charles P., aged twenty, butcher, already previously the subject of acute rheumatism, was admitted on December 8, 1864, the fourteenth day of his illness, and discharged on January 18, 1865. Four blisters, equal to 118⅝ square inches, were applied simultaneously, and with such relief that on the day succeeding the application all pain had disappeared, and his sleep, which for fourteen days had been wretched and without benefit to him, is reported as being good, and continuing satisfactory for the remainder of the time he was under observation. The pulse, which was 80 and 66 respectively on the two days before blistering, became 72, 68, 63, and ultimately 60. The temperature, 100.4 and 98.8 before blistering, became 98.8, 99.2, 98, and ultimately, on cure, 98.4. The heart was free from disease on admission, and continued the same during the entire period. The urine presented after the blistering, a very slight trace of albumen. The appetite, which had been bad since November 25, was very much improved on the day after the blisters had been applied. The report says, "he eats everything before him;" and it continued excellent from that date. The thirst was great on admission, only slight after the blisters were applied, and stated to be *nil* on the following day.

CASE 5.—Susan J., aged twenty-two, needlewoman, of fair complexion and sanguine temperament, and who had had three previous attacks, was admitted with acute rheumatism on December 12, the fourth day of her illness, and discharged on February 2, 1865. Six blisters were applied; four on December 12, and two on December 14, equal to 328 square inches. On the day succeeding the first application, the report states: "There is no pain now in the hands and ankles, but pain came on in the night in both shoulders, and she has had a very slight pain in passing urine." After the second application the report continues: "Feels much better; no pain anywhere; got out of bed this morning; wrist still swollen and red, but soft, movable, and not tender to pressure." On the following day report states: "She cannot even invent a pain anywhere; looks well, and is only weak. The pulse fell in this case from 114 to 100, 90, and 70, and was

84 at the time she left the Hospital. The temperature fell from 101.7 to 99.7, or two degrees. The heart was diseased on admission, and the systolic murmur at base and apex remained unchanged. The urine, at first acid, and containing a slight trace of albumen from the blisters, became alkaline on the second day from the time of the local application, and normal speedily after this date. Her sleep, which had been almost absent from December 8, is stated to have somewhat returned after the blisters were removed, to be much better on the fourth, and very good on the fifth night, and to have continued satisfactory until the time she was discharged. Her appetite, none on admission, was improved immediately after the application of the second set of blisters, and pronounced good on the fifth day after the last-named application. The thirst was very great on admission, unaltered the day after the blistering process, much less in amount on the following day, and none on the succeeding days.

The following case occurred under Dr. Davis's own care, at the London Hospital:

A case at present under the care of Dr. Davis affords an excellent example of the safe and rapid results of the treatment. The patient was brought into the London Hospital on Friday night completely crippled from acute rheumatism, and presenting the usual symptoms of that disease. He described his agony as intense. Ten blisters were at once applied, and a grain of morphia was administered internally.

On the Saturday evening he was free from all pain.

On the Sunday he declared himself to be comfortable, and his joints to be movable.

On the Monday morning he sat up and washed himself, and in the evening was able to walk down the ward to the closet.

On Tuesday he was perfectly easy, with appetite returned, and thirst absent; and was ordered to have a chop and a pint of Bass's ale, as he had been accustomed to live rather freely. The urine in this case was found to be strongly alkaline on re-action, and perfectly free from any trace of albumen as tested by heat and nitric acid. He had had no stranguary. On being questioned as to the pain induced by the blisters, he said that he would gladly have them repeated were he unfortunate enough to have another attack of rheumatism. No cardiac mischief was developed. No medicine beyond the morphia already mentioned had been administered.—*Medical Times and Gazette, April* 1, 1865, *p.* 333.

